# Semi-Annual Meeting of the Erie Co. Med. Society

**Published:** 1845-07-01

**Authors:** 


*Semi-annual meeting of the Erie Co. Med. Society.*—The Semi-annual meeting of the Erie Co. Med. Society, was held at the lecture room of the Young Mens' Association, in this city, on the 10th, ult. The Physicians of the city generally were present, but the attendance from the country was unusually small. A committee appointed to take into consideration measures to render the meetings of the Society more interesting, reported in favor of assembling on the next annual meeting at 10 A. M., continuing in session until the business is completed, and then adjourning to some appropriate place and *dining* together. A resolution was adopted to this effect. A committee of five were appointed to report at the annual meeting on the subject of medical legislation, education, &c. A patient with a large tumour on the occiput (probably aneurismal,) presented himself for examination. An agent for Dr. Jarvis' apparatus for the reduction of dislocations, and the treatment of fractures, illustrated and explained their application to the society. It was voted to supply each member of the society with a copy of the transactions of the meeting of the state society for the year 1845, and to subscribe for six copies of the Buffalo Medical Journal, one to be retained in the library, and the remainder to be presented to other county Societies.

The address was by H. M. Congar, M. D. His subject was the importance of popular instruction in Physiology, and the duty of medical men to labor for this object. The various considerations which render this object desirable, and the claims which it has upon members of the profession, were presented with much force and ability.

John S. Trowbridge, M. D., was appointed orator for the next meeting.

